# Dynamic Resolution of Coronary Vasospasm: The Clasp-Knife Sign

**DOI:** 10.1016/j.jaccas.2026.109120

**Published:** 2026-07-14

**Authors:** Ali Sezgin, Hacı Ali Kurklu, Ayse Avara, Oguz Ucar, Hakan Gullu

**Affiliations:** Department of Cardiology, Ankara Etlik City Hospital, Ankara, Türkiye

**Keywords:** clasp-knife sign, coronary angiography, coronary vasospasm, nitroglycerin, STEMI mimic

## Abstract

**Background:**

Coronary vasospasm may mimic acute coronary occlusion and ST-segment elevation myocardial infarction, potentially leading to unnecessary interventions if not recognized.

**Case Summary:**

A 32-year-old woman with no prior medical history presented with acute chest pain and inferior ST-segment elevation. Emergency coronary angiography demonstrated apparent complete occlusion of the proximal right coronary artery with absent distal flow. Before any mechanical intervention, intracoronary nitroglycerin was administered, resulting in abrupt restoration of vessel patency with normalization of luminal caliber and TIMI flow grade 3. This dynamic change created a characteristic appearance resembling the sudden opening of a clasp knife. The patient was managed conservatively with vasodilator therapy and remained clinically stable.

**Discussion:**

This case highlights a distinctive angiographic manifestation of coronary vasospasm. Recognition of this phenomenon, termed the clasp-knife sign, may help differentiate vasospastic occlusion from thrombotic coronary obstruction and prevent unnecessary coronary intervention.

## Visual Summary

**Figure:**
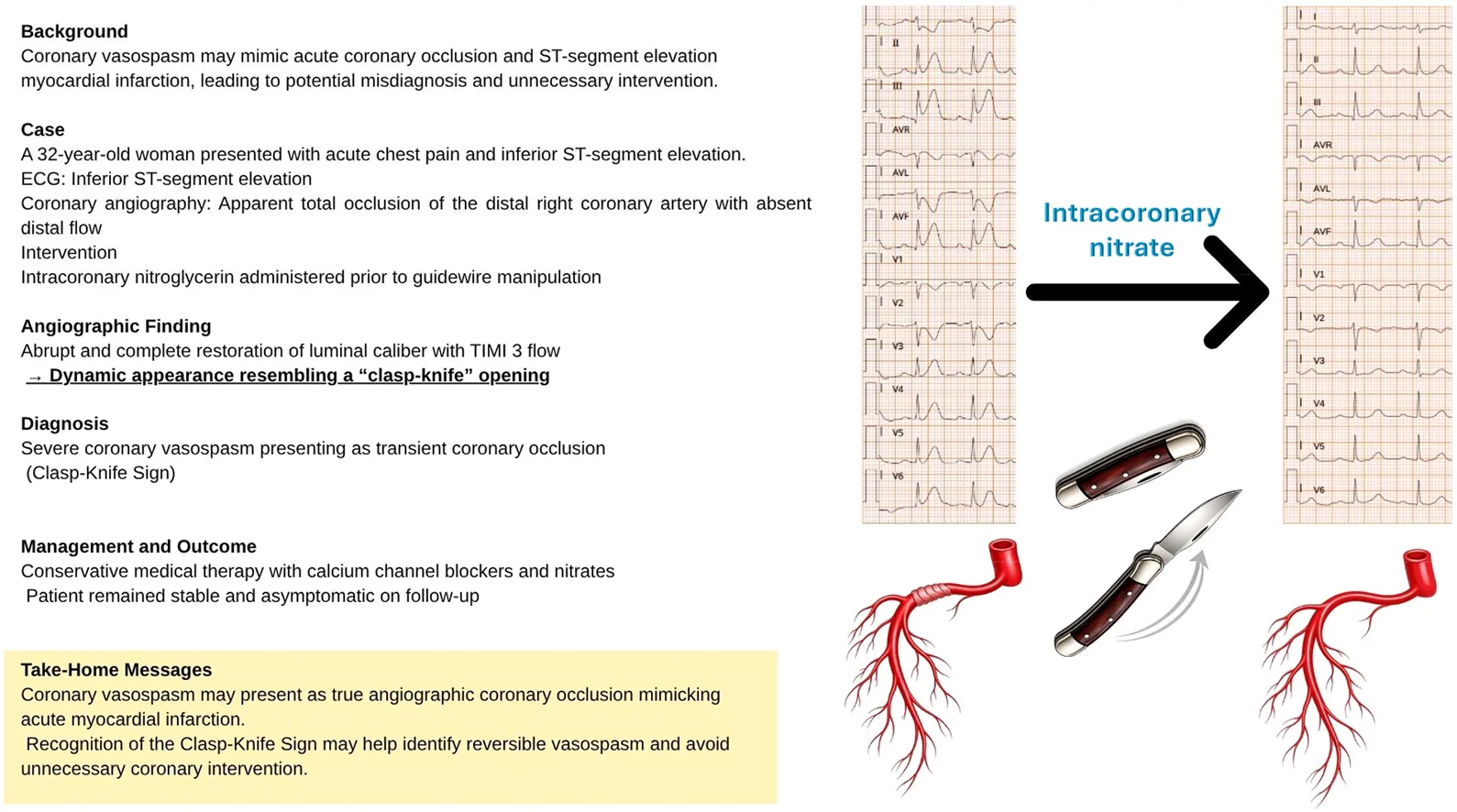

## History of Presentation

**Figure 1:**
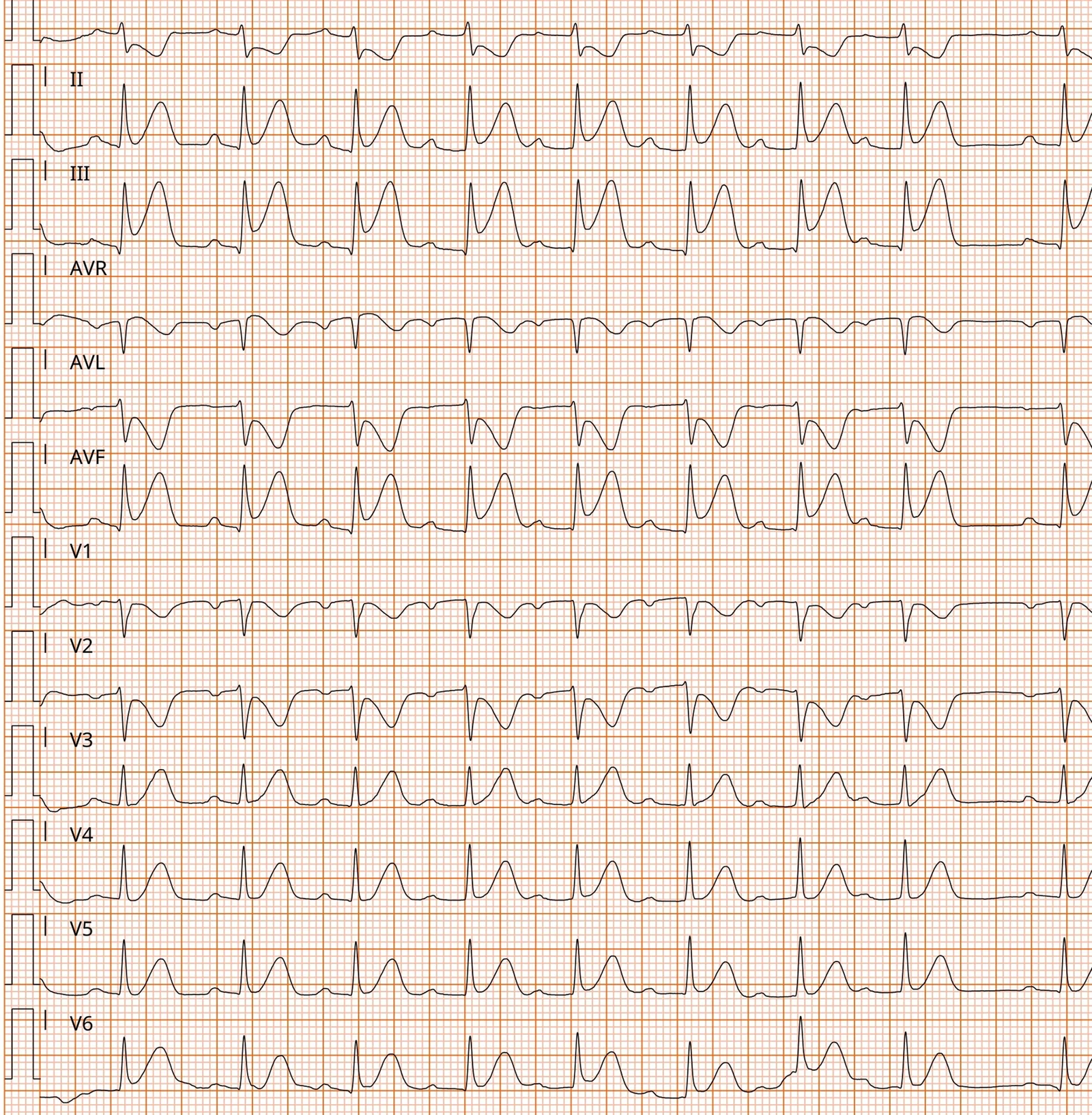
Initial Electrocardiogram at Presentation Electrocardiogram demonstrating ST-segment elevation in the inferior leads, consistent with inferior myocardial ischemia at presentation.

A 32-year-old female health care worker presented with acute-onset retrosternal chest pain radiating to the left arm. The symptoms began at rest and persisted for approximately 30 minutes. Initial electrocardiogram (ECG) demonstrated ST-segment elevation in the inferior leads, consistent with acute inferior myocardial infarction (Figure 1). The patient was urgently transferred to our center for primary coronary angiography.

On admission, the patient continued to experience chest discomfort. Vital signs were stable, and physical examination revealed no signs of heart failure. Cardiac auscultation was unremarkable.

## Past Medical History

The patient had no prior history of coronary artery disease, vasospastic angina, or nitrate-responsive chest pain and was not taking any regular medications. She reported no smoking history, recent emotional stressors, stimulant exposure, or prior episodes suggestive of vasospasm.

## Differential Diagnosis

Based on the patient's clinical presentation and ECG findings consistent with inferior ST-segment elevation myocardial infarction (STEMI), several potential diagnoses were considered. The primary consideration was acute thrombotic occlusion of the right coronary artery. However, alternative causes including coronary vasospasm, spontaneous coronary artery dissection, and coronary embolism were also included in the differential diagnosis.

## Investigations

Emergency coronary angiography was performed via femoral access. The left coronary system, including the left anterior descending and circumflex arteries, was angiographically normal.

**Figure 2:**
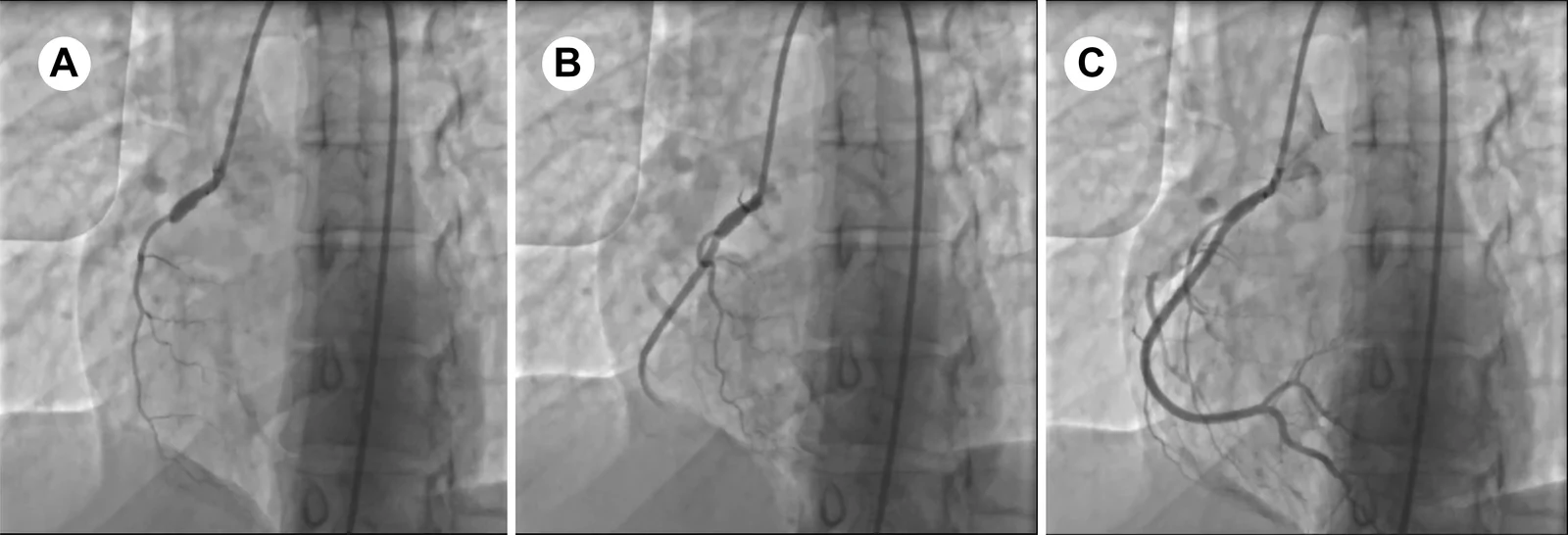
Frame-by-Frame Angiographic Demonstration of Clasp-Knife Sign (A) Baseline angiography demonstrating apparent right coronary artery occlusion distal to the right ventricular branch with absent distal flow. (B) Abrupt resolution of coronary vasospasm following intracoronary nitrate administration, with restoration of antegrade flow. (C) Complete restoration of distal right coronary artery flow without residual fixed stenosis. The rapid directional reopening of the vessel, visually resembling the unfolding motion of a clasp knife, defines the clasp-knife sign.

**Figure 3:**
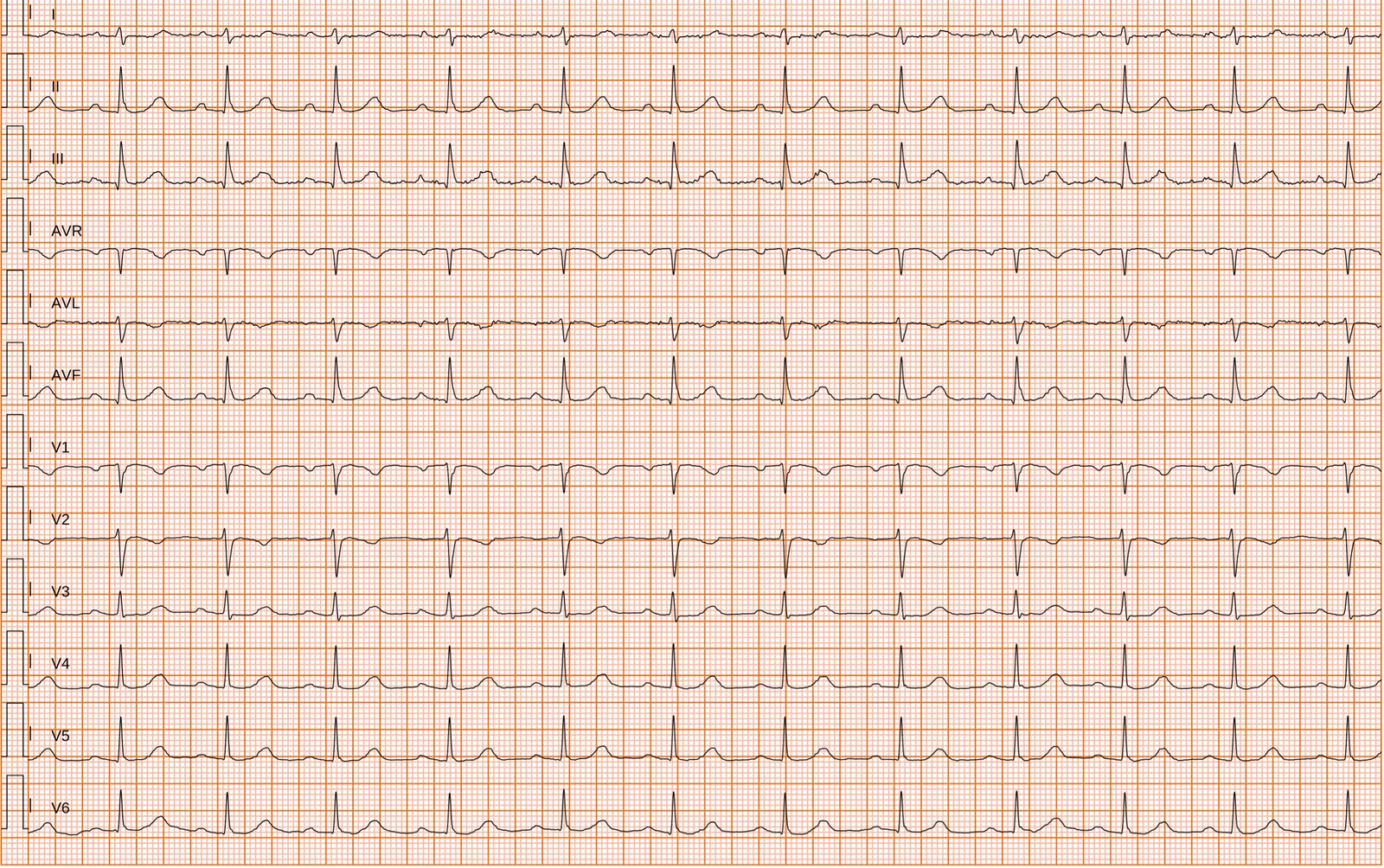
Post-Procedural Electrocardiogram Electrocardiogram demonstrating complete resolution of inferior ST-segment elevation following nitrate-induced relief of coronary vasospasm.

Selective angiography of the right coronary artery demonstrated complete occlusion of the proximal segment with absent distal flow (TIMI flow grade 0) (Video 1). During contrast injection, however, the vessel abruptly and completely reopened without any guidewire manipulation or mechanical intervention. The distal vessel became fully patent with restoration of TIMI flow grade 3 (Video 2, Figures 2A and 2B). This sudden transition from complete occlusion to a normal luminal diameter produced a striking angiographic appearance resembling the rapid opening of a clasp knife. Following restoration of normal coronary blood flow, complete resolution of ST-segment elevation was observed (Figure 3).

## Management

Given the angiographic appearance of complete distal occlusion, an initial strategy for percutaneous coronary intervention was considered. Intravenous unfractionated heparin was administered according to standard protocol. A Judkins Right 4 guiding catheter was carefully engaged into the right coronary artery.

However, considering the possibility of coronary vasospasm and to avoid further aggravation of vasoconstriction, intracoronary nitroglycerin (200 μg) was administered before attempting to cross the lesion with a guidewire. Following nitrate administration, the vessel demonstrated rapid and spontaneous resolution of the occlusion with restoration of normal luminal caliber and TIMI flow grade 3, confirming the diagnosis of severe coronary vasospasm.

In light of the complete resolution and absence of residual stenosis, mechanical intervention was deferred. The patient was subsequently managed conservatively with calcium channel blockers and nitrates.

## Outcome and Follow-Up

The patient's chest pain resolved completely following the procedure. Repeat electrocardiography demonstrated resolution of ST-segment elevation (Figure 2C). Cardiac biomarkers showed only mild elevation.

The patient remained hemodynamically stable throughout hospitalization and was discharged on medical therapy including calcium channel blockers and nitrates. At follow-up, the patient remained asymptomatic without recurrent angina or rehospitalization, supporting a favorable short-term clinical course.

## Discussion

Coronary vasospasm may closely mimic acute coronary syndromes, including STEMI, and in severe cases can manifest as apparent coronary occlusion with absent distal flow during emergency angiography. The primary pathophysiology involves transient coronary smooth muscle hyperreactivity leading to reversible vasoconstriction.^1^ This diagnostic overlap may lead to unnecessary intervention if vasospasm is not promptly recognized.^2^

A distinctive feature of this case was the abrupt restoration of vessel lumen, producing an angiographic appearance resembling the sudden opening of a clasp knife after intracoronary nitrate. We propose the term clasp-knife sign to describe this phenomenon, reflecting the rapid and dynamic transition from near-total occlusion to full patency.

In contrast to previously described angiographic manifestations of coronary vasospasm, such as focal narrowing, reversible occlusion, or string-of-beads appearance, the clasp-knife sign refers not only to the presence of spasm, but also to its characteristic dynamic resolution pattern following intracoronary nitrate administration. Indeed, underlying mechanisms do not differ among all vasospasm patterns. The hallmark feature is a rapid, directional reopening of an apparently occluded coronary segment with restoration of distal flow, visually resembling the unfolding motion of a clasp knife on cineangiography.

This dynamic resolution also strongly emphasized the importance of vasodilator administration before intervention. This case underscores the importance of administering intracoronary nitrates before guidewire manipulation when vasospasm is suspected.^3^ Prompt angiographic resolution after nitrate administration strongly favors a functional rather than structural obstruction and may prevent unnecessary coronary intervention. Because mechanical instrumentation can aggravate vasoconstriction and overestimate lesion severity, early nitrate use should be considered before wiring in angiographically ambiguous occlusions.

From a practical perspective, several clinical and angiographic red flags may raise suspicion for coronary vasospasm during emergency angiography and help avoid unnecessary intervention. Patient-related clues include younger age, female sex, limited traditional cardiovascular risk factors, prior episodes of rest angina, smoking, emotional stressors, or stimulant exposure (eg, cocaine or amphetamines). Angiographic clues include abrupt coronary occlusion without clear thrombotic features, lesion severity disproportionate to the clinical presentation, preserved distal vessel silhouette despite apparent proximal obstruction, dynamic lesion morphology, and rapid angiographic improvement after intracoronary nitrate administration. In such situations, early nitrate administration before guidewire manipulation may help distinguish functional vasoconstriction from fixed coronary obstruction and reduce the risk of unnecessary stenting.

Although the angiographic findings and immediate response to intracoronary nitrate strongly supported vasospasm, additional diagnostic considerations merit discussion. Intracoronary imaging was deferred because the acute STEMI setting and complete angiographic resolution after nitrate administration substantially reduced diagnostic uncertainty. However, in a nonemergent setting, intracoronary imaging modalities such as optical coherence tomography could have provided complementary mechanistic information by excluding subtle plaque disruption or other structural abnormalities.^4^ Similarly, invasive provocative testing was not pursued, as spontaneous angiographic resolution after vasodilator therapy rendered further provocation clinically unnecessary in the acute setting.^5^

## Conclusions

In angiographically ambiguous occlusions suspicious for vasospasm, administration of an adequate dose of intracoronary nitrate before guidewire manipulation, followed by slightly prolonged cine acquisition, may help reveal dynamic vasospastic resolution patterns. In the present case, this characteristic opening movement, described as the clasp-knife sign, became apparent during nitrate-induced restoration of coronary flow and may represent a practical visual clue supporting vasospasm.

**Visual Summary:**
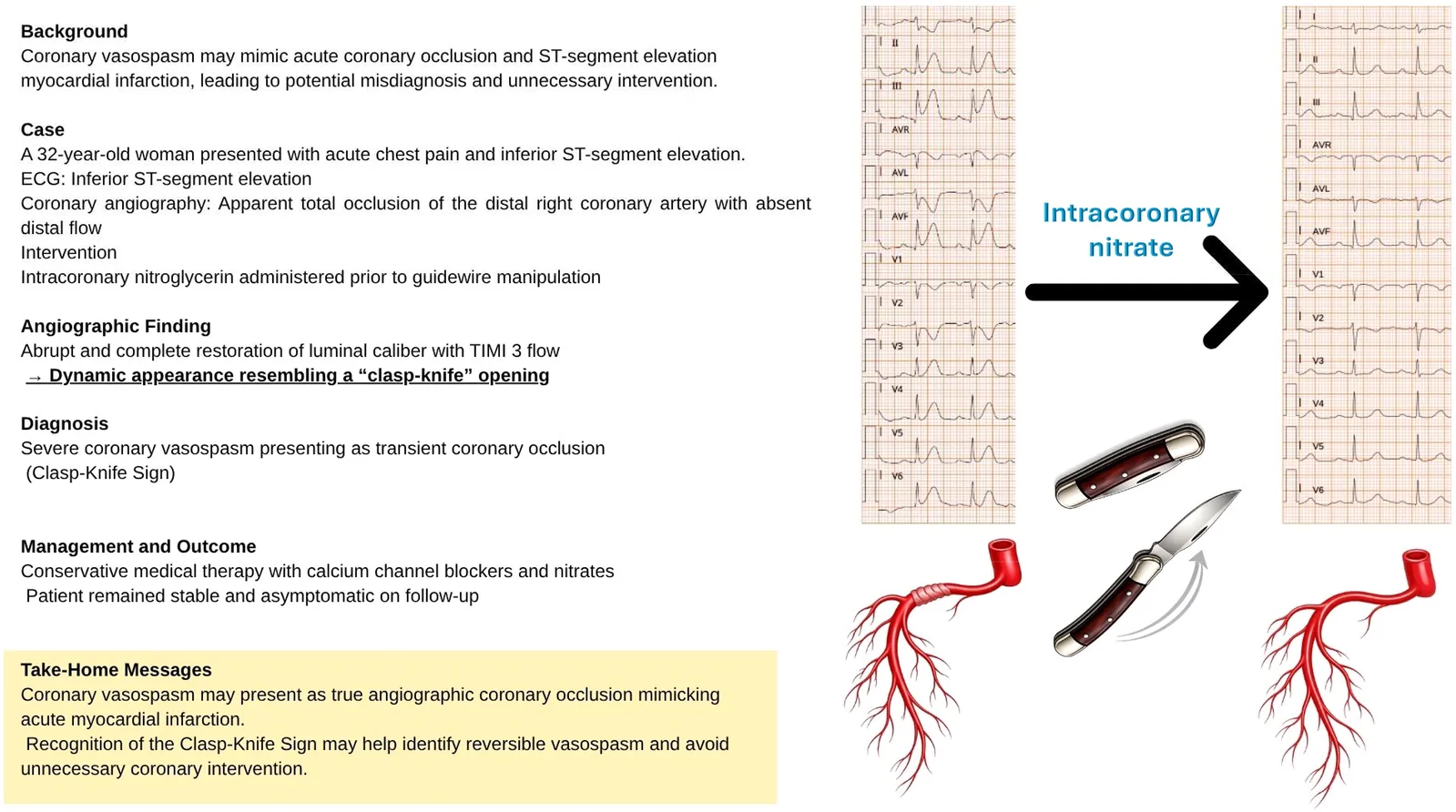
Abrupt Opening of the Coronary Artery Like a Clasp-Knife The diagnostic challenge of coronary vasospasm manifesting as apparent coronary occlusion, the role of early intracoronary nitrate administration before guidewire manipulation, and the dynamic nitrate-induced resolution pattern described as the clasp-knife sign. ECG = electrocardiogram.

## Funding Support and Author Disclosures

The authors have reported that they have no relationships relevant to the contents of this paper to disclose.

## Appendix

Video 1 Vasospasm-Induced Coronary Occlusion Coronary angiography in the left anterior oblique projection demonstrating apparent total right coronary artery occlusion caused by severe vasospasm, with absent distal flow.

Video 2 Dynamic Resolution of Clasp-Knife Sign Coronary angiography demonstrating nitrate-induced resolution of vasospasm without guidewire manipulation, revealing the characteristic dynamic reopening pattern described as the clasp-knife sign in the left anterior oblique projection.

## References

[1] Khan K., Luu J.M. (2026). Severe coronary vasospasm and the role of nontraditional risk factors. JACC Case Rep.

[2] Ralota K.K., Layland J. (2025). Vasospastic angina: pathophysiology, diagnosis, and emerging therapeutic approaches. World J Cardiol.

[3] Soliman D., Castillo-Rodriguez C., Cruz D., Abdelmalek J. (2025). A Rare Case of Nitroglycerin∖ Resistant Coronary Arterial Vasospasm During Coronary Artery Bypass Surgery. Catheter Cardiovasc Interv.

[4] Abdu F.A. (2026). Myocardial infarction with non-obstructive coronary arteries: a multimodal imaging approach to diagnosis and etiology-targeted therapy. Curr Probl Cardiol.

[5] Savulescu-Fiedler I., Baz R.O., Baz R.A., Scheau C., Gegiu A. (2025). Coronary artery spasm: from physiopathology to diagnosis. Life (Basel).

